# Enhanced Coarse-Grained WC-Co(Ce) Cemented Carbide Prepared through Co-Precipitation

**DOI:** 10.3390/ma16165506

**Published:** 2023-08-08

**Authors:** Fanlu Min, Shiyu Wang, Songbai Yu, Hao Yang, Zhanhu Yao, Jianzhong Ni, Jianfeng Zhang

**Affiliations:** 1 Key Laboratory of Geomechanics and Embankment Engineering, Hohai University, Ministry of Education, Nanjing 210024, China; 2 College of Civil and Transportation Engineering, Hohai University, Nanjing 210024, China; s1346728159@163.com; 3 College of Mechanics and Materials, Hohai University, Nanjing 211100, China; yulansongbai@163.com (S.Y.); jfzhang@hhu.edu.cn (J.Z.); 4 State Grid Lu’an Electric Power Supply Company, Lu’an 237000, China; yanghao__yh@163.com; 5 CCCC Tunnel Engineering Company Limited, Beijing 100102, China; yaozhanhu321@163.com; 6 Hangzhou Fuyang City Construction and Investment Group Company Limited, Hangzhou 311400, China; fyctnjz@yeah.net

**Keywords:** chemical co-precipitation, influence law, carbides, mechanical properties, micromorphology

## Abstract

The exploration of coarse-grained WC cemented carbide has become a research hotspot for its application in the fields of rock cutting and mining; a key issue is how to achieve uniform dispersion and densification of the sintered phase, as well as how to obtain better mechanical properties. In this paper, chemical co-precipitation, combined with hydrogen reduction, was adopted. CoCl_2_·6H_2_O and CeCl_3_ were used as precursors to coat Co nanoparticles on the surface of WC powder while introducing different contents of cerium; the samples were then sintered and densified to obtain WC-Co(Ce) hard alloy materials. On the surface of the obtained WC particles, the distribution of Co(Ce) nanoparticles was uniform and dense, and the average particle size after sintering was 4.2 μm, which lies in the coarse-grained range. The addition of cerium elements significantly improves the flexural strength and impact toughness; when the cerium content was 0.5% and 0.6%, they increased to 2487 MPa and 36.1 kJ/m^2^, respectively. The addition of Co(Ce) through the co-precipitation method could achieve a uniform coating of the Co phase, along with the uniform dispersion and densification of the sintered phase, giving the WC-Co(Ce) cemented carbide excellent properties.

## 1. Introduction

Tungsten carbide-cobalt (WC-Co) cemented carbide is formed by composite of tungsten carbide (WC) and cobalt (Co) through powder metallurgy. Its abrasiveness and impact toughness have attracted the attention of researchers [1,2,3,4], and it has been widely used in machining tools, rock cutting, mining, underground shield engineering, and other related fields [5,6,7]. In particular, coarse-grained cemented carbide occupies a prominent position in rock cutting and mining because of its high hardness and good impact toughness [4]. However, the sintering of coarse-grained WC-Co cemented carbide is restricted by the size effect and surface energy effect, which makes the microscopic structure and mechanical performance of the alloy unable to meet current requirements [8]. Kezhong et al. [9] used WC and Co with Fischer particle technology and densification technology to prepare cemented carbide for shield cutters 16–28 μm in size as raw materials, and utilized phase transformation control technology and densification technology to prepare cemented carbide for shield cutters, further analyzing their engineering properties. The coarser the WC grain, the better the tool toughness and fatigue resistance strength. Mingyuan et al. [10] studied the effect of the ratio of 5μm coarse grain and 1μm fine grain WC on the microstructure and mechanical properties of low-cobalt cemented carbide, and the hardness of the alloy decreased and the fracture toughness increased with the increase in the coarse-grained WC content. Bin et al. [11] prepared WC-15Fe-5Ni cemented carbide with different grain sizes and found that with a larger grain size, the internal defects of the alloy increased and the fracture was dominated by the grain penetration mode. In the current research, when preparing coarse-grained or ultra-coarse cemented carbides, due to the coarse initial grains, grain fragmentation defects easily occur at the boundary of the binder phase, making the material prone to poor wettability and grain growth issues, which deteriorates the mechanical properties of the composites [12,13,14].

The uniform mixing of the powder is a prerequisite for improving these problems and for the preparation of high-performance cemented carbides. The hydrothermal pressure hydrogen reduction method [15], electroless plating method [16], and chemical co-precipitation method [17] are the most commonly used, and the powder prepared through these methods is relatively uniform, with a more consistent particle size. In particular, the chemical co-precipitation method reacts metal compounds and precipitants in solution to obtain metal precipitates [18], which has many advantages, including a simple preparation process, easy parameter adjustment, short reaction time, and low product impurity content. Yexi et al. [19] combined the cobalt powder preparation process with the powder mixing process, prepared WC-Co coated powder using the chemical co-precipitation method, and successfully prepared WC-8%Co ultra-coarse cemented carbide with good grain size controllability, a uniform structure, and excellent performance, which helps to overcome the issues regarding the particle size of WC being significantly reduced during the mixing process of the traditional wet grinding process. Yuxiang et al. [20] used ammonium tungstate and cobalt nitrate as raw materials, calcined them after chemical precipitation, and then directly reduced and carbonized them to obtain ultrafine WC-Co composite powder in a vacuum atmosphere. This method has a low raw material cost, simple process, and can easily be controlled.

Previous studies have shown that the addition of cerium elements can strengthen the binder phase, improve the wettability of the binder relative to the carbide phase, and refine the hard phase structure, thereby improving the flexural strength, impact toughness, and durability of cemented carbide [21,22,23]. At present, rare earth elements are primarily mixed with other alloy powders in the form of solid powder. Due to the small addition amounts, it is difficult to ensure the uniform dispersion of the powder when using the ball milling method, which makes it difficult to effectively improve the performance of cemented carbide. Xiaoliang et al. [24] prepared WC-10%Co-5%Ni cemented carbide using the co-precipitation method with the addition of rare earth Ce by means of intermediate alloy Ni-Ce. In comparison to an alloy prepared through traditional ball milling, the co-precipitation method can make the distribution of rare earth in the alloy more uniform. As a result, the fracture strength, impact toughness, and wear ratio of cemented carbide are increased to 1918 MPa, 200 J, and 30.21, which are 15.8%, 21.2%, and 47.9% higher than those of the traditional ball milling method, respectively. However, the effect of Ce on the dispersion behavior and mechanical properties of WC-Co remains to be studied in full.

In this study, Co and Ce salts were coated on the surface of WC powder using the chemical co-precipitation method, and the WC-Co(Ce) coated powder was then obtained through high-temperature hydrogen reduction. The influence of different process conditions—such as the solution concentration, reaction temperature, and precipitant addition time—on the powder precipitation rate and coating morphology were analyzed. The effect of the addition of rare earth element on the microstructure, densification behavior, and properties of cemented carbide is discussed, and the mechanism of the powder coating and performance enhancement is analyzed.

## 2. Experimental Procedure

### 2.1. Powder Preparation and Sintering

#### 2.1.1. Powder Preparation

CoCl_2_·6H_2_O (purity ≥ 98.0%, Shanghai Lingfeng Chemical Reagent Co., Ltd., Shanghai, China), (NH_4_)_2_C_2_O_4_·H_2_O (purity ≥ 99.5%, Shanghai Lingfeng Chemical Reagent Co., Ltd., Shanghai, China), and CeCl_3_ (purity ≥ 99.99%, Shanghai Lingfeng Chemical Reagent Co., Ltd., Shanghai, China) raw materials were used to prepare CoCl_2_, (NH_4_)_2_C_2_O_4_, and CeCl_3_ solutions of different concentrations. Slowly add the coarsened WC powder (Zigong Cemented Carbide Co., Ltd., Zigong, China) into the CoCl_2_ solution, and fully stir the mixture with a stirrer at a speed of 300 r/min for 20 min. Then, evenly add the precipitant (NH_4_)_2_C_2_O_4_ solution drop-wise at a certain rate into the mixed solution of CeCl_3_, CoCl_2_, and WC powders, and after it is fully reacted, allow it to stand at room temperature for 30 min. The chemical co-precipitation reaction process is as follows:

CoCl_2_·6H_2_O + 4(NH_4_)_2_C_2_O_4_·H_2_O + 2CeCl_3_→CoC_2_O_4_·2H_2_O↓ + 8NH_4_Cl + 8H_2_O + Ce_2_(C_2_O_4_)_3_↓ (1)

The sedimentation rate is obtained using the following formula:

precipitation rate = M_a_/M_t_ (2)

M_t_ and M_a_ are the theoretical masses obtained after the co-precipitation reaction and the powder mass obtained after drying, respectively.

Filter and dry the standing mixed solution to obtain the WC-Co(Ce) precursor powder, reduce it at a high temperature in a hydrogen atmosphere, and finally obtain the WC-Co(Ce) composite powder. The chemical reaction process is as follows:

2CoC_2_O_4_·2H_2_O + Ce_2_(C_2_O_4_)_3_ + 8H_2_→2Co + 2CeO_2_ + 4CO_2_↑ + 6CO↑ + 10H_2_O (3)

**Figure 1:**
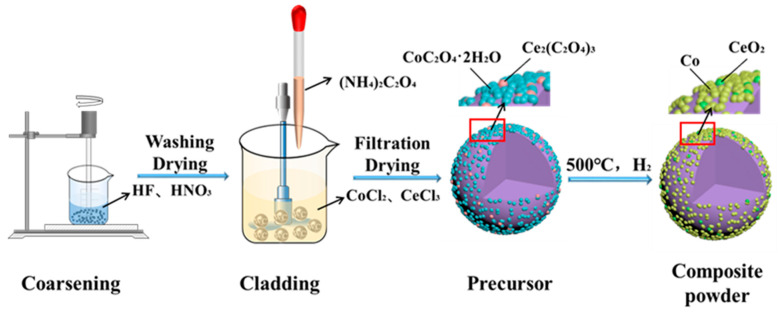
Flowchart of the preparation of WC-Co(Ce) composite powder by the chemical co-precipitation method.

The overall flowchart of the experiment for preparing the composite alloy powder through chemical co-precipitation is shown in Figure 1.

#### 2.1.2. Sintering

To analyze the effects of different cerium additions on the mechanical properties, grain size, and microstructure of the alloy, the above-mentioned experimental methods were used to determine the effects of WC additions of 0%, 0.2%, 0.3%, 0.4%, 0.5%, and 0.6% Ce. The powders were coated to obtain WC-Co(Ce) composite powders with different Ce contents. Then, 2.25 wt% of SD-E molding agent (origin: Zhuzhou, Hunan) was added and pelletized, respectively, followed by pressing and molding at 150 MPa, and finally, vacuum sintering in a vacuum furnace. During the sintering process, the furnace temperature was raised from room temperature to 200 °C at a rate of 3 °C/min, held for 0.5 h, then raised to 470 °C at a rate of 2 °C/min, held for 1.5 h, and then increased at a rate of 6 °C/min to 900 °C, held for 30 min, then raised to 1250 °C at a rate of 7 °C/min, held for 30 min, and finally raised to 1450 °C at 2.5 °C/min, and held for 1 h [25]. In the cooling stage, the temperature in the furnace was reduced to room temperature.

### 2.2. Microstructure and Performance Testing

The microstructure of the powder was observed using a ZEISS EVO18 scanning electron microscope (ZEISS, Jena, Germany); the D8 Advance X-ray powder diffractometer (Bruker, Karlsruhe, Germany) was used to determine the phase composition of the experimental samples. The working parameters of the X-ray diffractometer during the test were as follows: the X-ray tube voltage was 40 kV, the current was 40 mA, the test accuracy was ≤0.02°, the test rate was 10°/min, and the test angle was 5°–90°. The Olympus HX-MD50 (Tokyo, Japan) was used to obtain a metallographic photograph of the corroded alloy. The linear intercept method was used to measure the size of the WC grains falling on the straight line drawn on the SEM photo. Further, the measured grains were no less than 300, and the average grain size of the WC was determined. The Rockwell hardness was determined using the HRS150 type digital display Rockwell (Milwaukee, WI, USA) hardness tester for test [26]. The flexural strength was determined using an electronic universal testing machine (Sinter, Changchun, China) for testing [27]. The impact toughness was attested by JB-300 type manual pendulum impact tester (Fangyuan Testing Instrument Co., Jinan, China). (Values are means ± s.d).

## 3. Results and Discussion

### 3.1. Influence of Process Parameters on the Precipitation Rate

**Figure 2:**
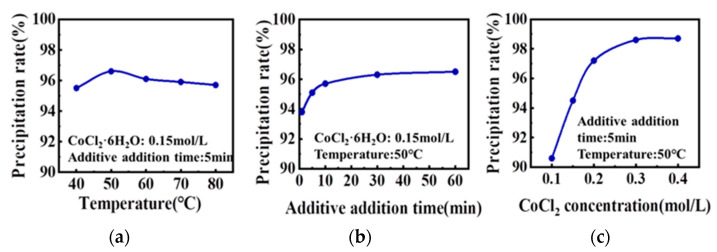
Influence of different process parameters on the precipitation rate of the co-precipitation reaction:(**a**) reaction temperature; (**b**) precipitant addition time; and (**c**) solution concentration.

Figure 2 shows the effect of the solution temperature, precipitant addition time, and solution concentration on the precipitation rate of the precursor. The optimum powder precipitation rate can be obtained when the process parameters are 50 °C, 30 min, and 0.3 mol/L. As shown in Figure 2a, when the temperature increased from 40 °C to 50 °C, the precipitation rate increased significantly, and the precipitation rate decreased slowly when the temperature was greater than 50 °C. At 80 °C, the precipitation rate was close to that at 40 °C. As shown in Figure 2b, when the addition time is within 10 min, the formation rate of the precipitation is faster; after 10 min, the growth rate is slower, and the precipitation rate tends to flatten after 30 min. As shown in Figure 2c, when the solution concentration is within 0.2 mol/L, the formation rate of the precipitation is faster; after 0.2 mol/L, the growth rate is slower, and the precipitation rate after 0.3 mol/L tends to be somewhat constant.

**Figure 3:**
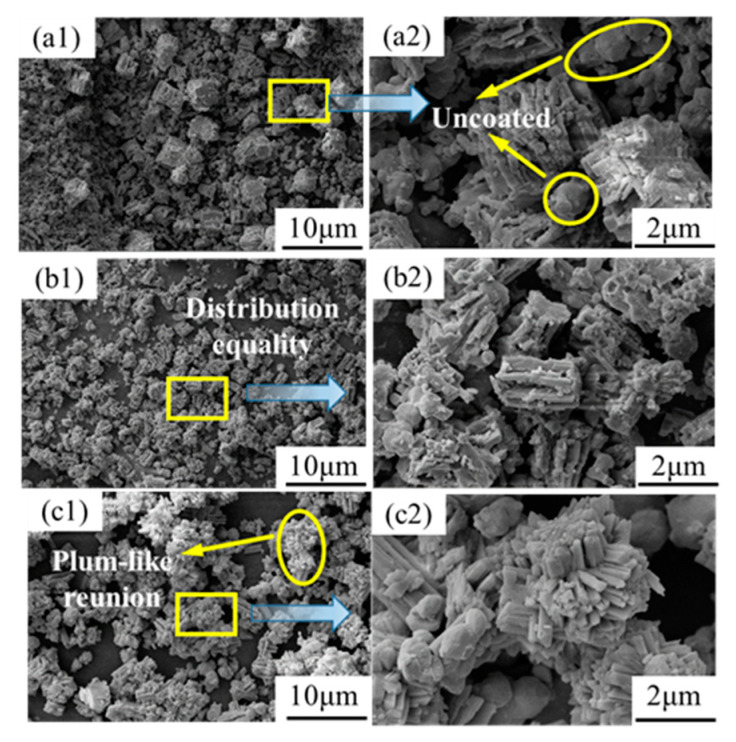
SEM images of the mixture precursor powder under different CoCl_2_ solution concentrations:(**a1**,**a2**) 0.1 mol/L; (**b1**,**b2**) 0.15 mol/L; and (**c1**,**c2**) 0.3 mol/L.

Figure 3 shows the SEM pictures of the mixture precursor powders at the solution concentrations of 0.1 mol/L, 0.15 mol/L, and 0.3 mol/L. When the concentration of the solution is low, the precursor powder contains plum-shaped cobalt-cerium oxalate, which has a large particle size, and some of the cobalt–cerium oxalate precursor powder is well attached to the WC particles. When the concentration of the CoCl_2_ solution further increases, the overall distribution of the precursor particles is uniform, most of the cobalt and cerium oxalate particles become particles with relatively large lengths and diameters, and only a small portion of the WC particles are not coated. When the solution concentration continues to increase further, plum-like agglomeration occurs, and the powder uniformity deteriorates.

During the precipitation process, if the rate of crystal growth is greater than that of the crystal nuclei, grains with relatively large lengths and grain sizes will be formed [28]. During the co-precipitation process, due to the low concentration of CoCl_2_ in the solution, the co-precipitation reaction in the solution is slow, and the instantaneously generated crystal nuclei are few; therefore, the crystal growth rate is fast, resulting in larger crystal particles of cobalt-cerium oxalate. When the concentration of CoCl_2_ in the solution increases, the co-precipitation reaction proceeds, the reaction speed is accelerated, the formation speed of the crystal nuclei is accelerated, the precipitation quickly reaches saturation, and the precipitation speed of the cobalt-cerium oxalate crystal becomes faster. This results in a lack of growth momentum of the generated cobalt–cerium oxalate crystals, and the length and particle size of the cobalt–cerium oxalate crystals generated by the co-precipitation reaction are relatively small. However, when the concentration of the CoCl_2_ solution in the solution continues to increase, the uniformity of the powder generated by the solution becomes poor, and some crystal nuclei in the solution will collide with each other, causing the crystals to develop together, resulting in agglomeration. Finally, the length and particle size of the cobalt–cerium oxalate crystals produced by the co-precipitation reaction become larger.

### 3.2. Phase Composition and Analysis of the Powder Coating Effect

**Figure 4:**
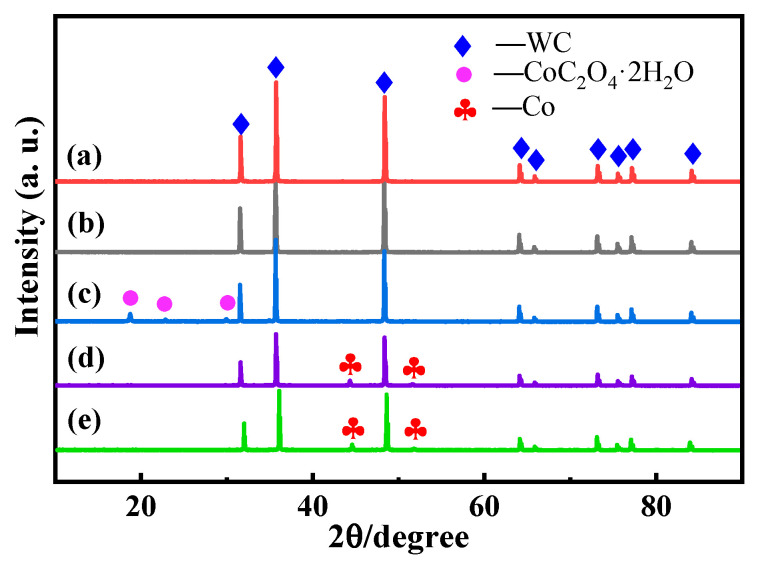
XRD patterns of mixed powders at different stages prepared by chemical co-precipitation: (**a**) WC powder; (**b**) WC powder after coarsening; (**c**) mixed precursor powder; (**d**) powder without cerium reduction; and (**e**) powder after reduction by adding 0.2% cerium.

Figure 4 shows the XRD patterns of the composite powders at different stages prepared through chemical co-precipitation. From (a) and (b), there are no obvious impurities in the original WC powder, and the roughened powder will not change its elemental composition due to the roughening liquid. Diffraction pattern (c) proves that the powder obtained via the chemical co-precipitation reaction is a mixture of WC and CoC_2_O_4_·2H_2_O, and no other impurity elements are generated, indicating that a relatively pure powder is obtained. Diffraction pattern (d) shows the XRD pattern of the mixture precursor without any cerium element addition after being reduced by high-temperature hydrogen, and the composite powder of WC and Co was obtained after the reduction in the precursor; in comparison to Diffraction pattern (a), no other impurity elements are generated. Diffraction pattern (e) is the mixture of the XRD patterns obtained after adding 0.2% cerium elements and the reduction using high-temperature hydrogen. The phases of the cemented carbides with different cerium contents comprise hard-phase WC and binder-phase Co. Cerium-containing phases were not detected. This may be due to the low content of cerium elements, which is lower than the detection limit of the equipment. This shows that adding a small amount of cerium elements does not change the phase of the alloy. Therefore, the feasibility of preparing WC-Co(Ce) composite powder using the chemical co-precipitation method was proven by the XRD diffraction pattern.

**Figure 5:**
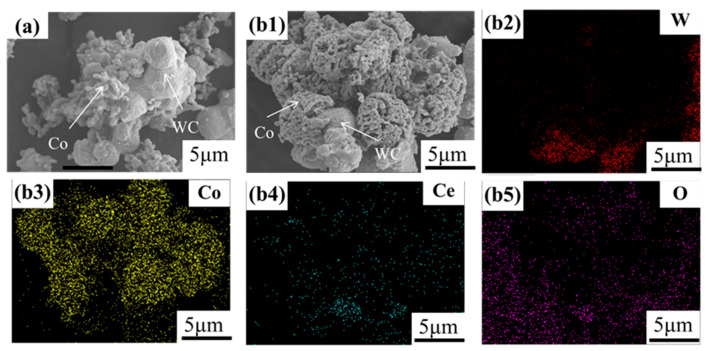
SEM image of the reduced mixture precursor powder: (**a**) Powder after reduction without adding cerium; (**b1**) powder after reduction with 0.6wt% cerium added; (**b2**–**b5**) are the elemental analysis images of (**b1**) for W, Co, Ce, and O, respectively.

Figure 5 shows a high-magnification SEM image of the reduced powder prepared using the co-precipitation method. The particle size of the Co phase in the coated powder is at the micron or submicron level. Without the addition of cerium elements, the Co phase is attached to the surface of the WC particles in a coarse dendrite shape, but the contact area of the WC particles is small and the coating effect is poor. After the addition of cerium, the morphology of the Co phase changed greatly, showing uniform, dense, and regular layer-like accumulation, as well as high adhesion and a good encapsulation effect with the WC particles [29]. According to Figure 5b, the addition of cerium elements results in less exposed parts of W, and Co and Ce are uniformly coated on the surface of the WC particles. The high content of oxygen elements in the lower left corner of Figure 5(b5) is due to the presence of oxygen elements in the substrate carrying the sample.

### 3.3. Cemented Carbide Microstructure

**Figure 6:**
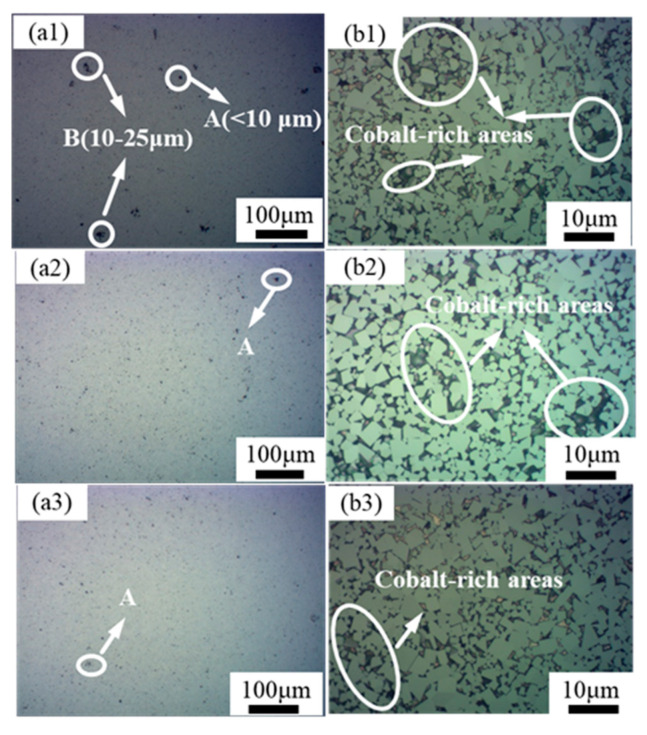
Metallographic diagram of WC-Co(Ce) with different cerium elements contents: (**a1**,**b1**) 0 wt% Ce; (**a2**,**b2**) 0.3 wt% Ce; (**a3**,**b3**) 0.6 wt% Ce.

The abrasive surfaces of the WC-Co(Ce) cemented carbide samples with different cerium contents made into mirror surfaces were corroded with reagents. Metallographic photographs of cemented carbide at 100× and 1000× magnification are shown in Figure 6. When the cerium elements content is 0 wt%, the porosity level is B04. With the gradual increase in cerium content, the gas in the alloy is discharged and the porosity gradually decreases. When the cerium elements content is 0.6 wt%, the pores in the metallographic diagram of cemented carbide are the least, and the porosity level is A04. It can also be seen from the high-magnification metallograph in Figure 6b that the cobalt-rich region decreases and the cobalt phase is less enriched with the increasing cerium content, which implies a more homogeneous distribution of the Co phase.

**Figure 7:**
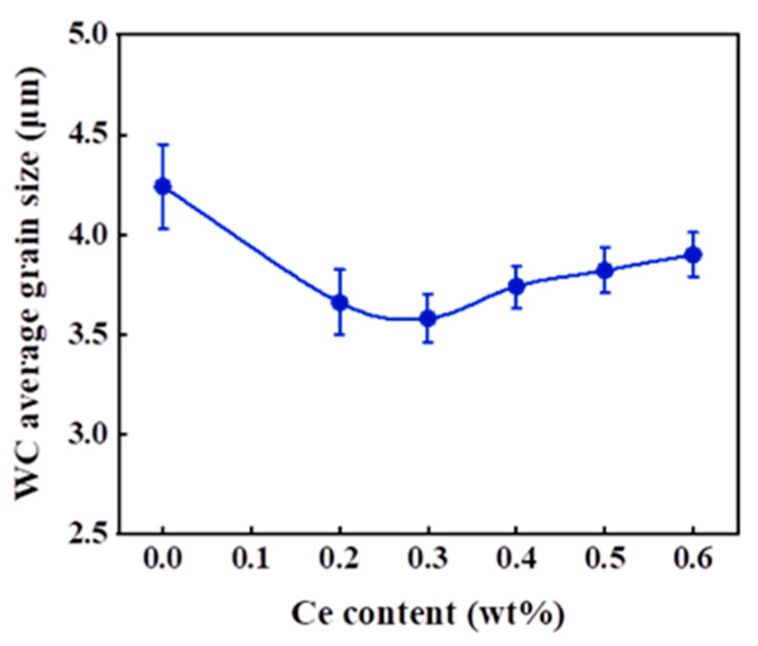
Effects of different cerium contents on the average grain size of WC-Co(Ce).

The WC grain sizes in alloys with different cerium contents prepared through the chemical co-precipitation process were measured according to the truncated grain method, and the average grain statistics are shown in Figure 7. The alloys prepared via the chemical co-precipitation process belong to the category of coarse-grained cemented carbide (the WC grain size is 2.5–5.0 μm). With the increase in the cerium content, the WC grain size decreases and then increases. When the cerium addition is 0.3 wt%, the average grain size is the smallest, 3.58 μm, which is slightly smaller, by about 0.5 μm, compared with the WC prepared without cerium addition. The decrease in error bars indicates that the WC grain size distribution is reduced and the addition of cerium elements can make the WC grain size more uniform.

### 3.4. Mechanical Properties

#### 3.4.1. Relative Density and Rockwell Hardness

**Figure 8:**
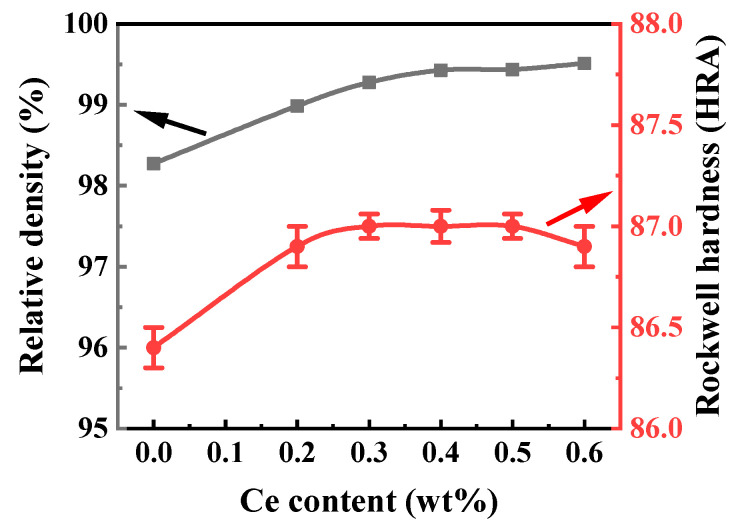
Variation of the relative density and Rockwell hardness of cemented carbide with Ce content.

From the relative density curve in Figure 8, when the addition of cerium elements is less than 0.4%, the relative density linearly increases with the increase in the cerium element content. The relative density of the alloy reaches 99.5% at 0.4%–0.6%. It is shown that under the same cobalt content, the addition of cerium elements is conducive to the distribution of the cobalt phase to evenly fill some pores and to improve the relative density of the cemented carbide.

The hardness test results are shown in Figure 8. With the increase in the Ce content, the Rockwell hardness of the alloy slightly increased. The Rockwell hardness of the alloy with 0.3 to 0.5 wt% Ce is 87 HRA, which is 0.6 HRA higher compared to the alloy without the addition of cerium. The hardness of the alloy is mainly a consequence of the hard-phase WC, and the cerium additives mainly strengthen the bonding force between the cobalt phase and the tungsten carbide phase by purifying the grain boundaries [30]. Therefore, the addition of cerium does not affect the hardness of the cemented carbide too much. The slight increase in the hardness is due to the increase in the relative density and the WC grains’ refinement caused by the cerium addition. And the hardness of Cemented Carbide shows an inverse correlation with the grain size, which leads to the decrease in the hardness at a 0.6 wt% cerium addition.

#### 3.4.2. Flexural Strength and Impact Toughness

**Figure 9:**
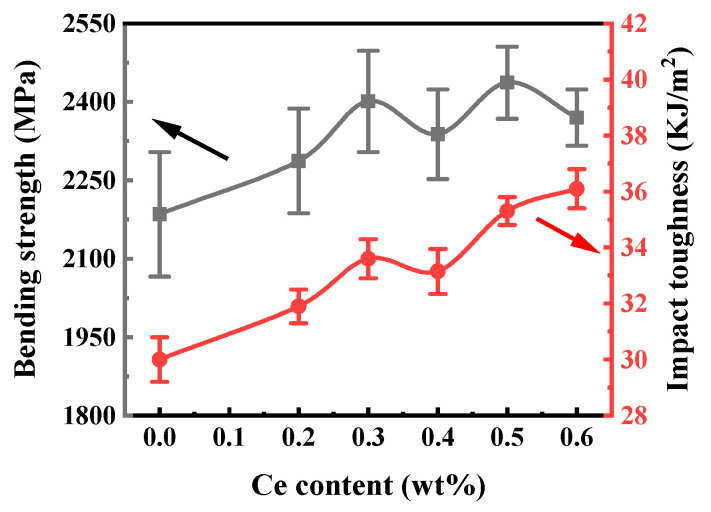
Effect of Ce content on the bending strength and impact toughness of cemented carbide.

The test results for the flexural strength and impact toughness of the cemented carbide are shown in Figure 9. When no cerium element is added, the average flexural strength of the WC-Co cemented carbide is 2185 MPa. When the amount of cerium added to the cemented carbide is 0~0.3%, the flexural strength increases with the increase in the cerium content. When the amount of cerium added into the cemented carbide continues to increase, the flexural strength of the cemented carbide is basically unchanged, showing a tendency to fluctuate in a range interval. When the addition amount is 0.5%, the average value of the flexural strength of the cemented carbide is 2487 MPa. It can also be seen that the impact toughness of the cemented carbide is positively correlated with the content of cerium, showing a fluctuating upward trend. The impact toughness of the cemented carbide reaches the maximum value of 36.1 kJ/m^2^ when its addition amount is 0.6wt%. Combining the above-mentioned variation rules, we can see that when the addition amount of cerium is 0.5%, the improvement in the flexural strength and impact toughness of the cemented carbide is the best.

## 4. Mechanism Analysis

**Figure 10:**
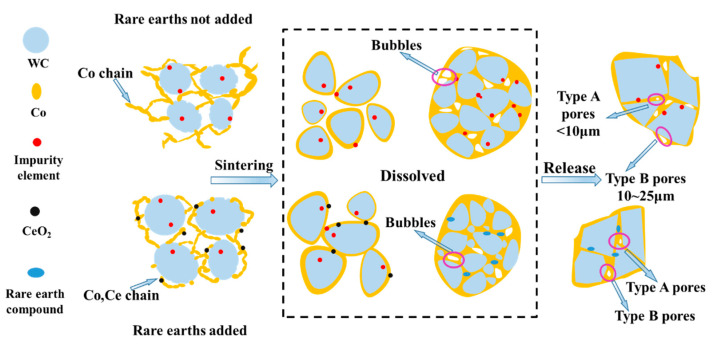
Influence mechanism of cerium elements on the dissolution and desorption of WC from Co during sintering.

When cerium is not added, cobalt oxalate grows along its own crystal surface due to the principle of lowest energy, so the cobalt phase shows dendritic shape and poor adhesion to the WC grains. After adding cerium, the cerium elements are selectively adsorbed on the surface of the WC particles, which reduces the energy on the surface of the WC particles, thus weakening the preferred growth of cobalt oxalate [31]. As a result, the cobalt oxalate particles can grow on different crystal surfaces; therefore, the cobalt phase presents a lamellar stacking body morphology. The presence of cerium elements improves the cobalt encapsulation effect on the WC particles and lays the foundation for the uniform dispersion of the cobalt phase in the subsequent sintering process. During the sintering process, with the increase in temperature, the cobalt phase melts into a liquid phase at a high temperature. WC is partially dissolved in Co, and the flow of the liquid phase between the original particles makes the defect position at the grain interface participate in the grain dissolution–precipitation growth process, realizing the liquid phase redistribution and particle rearrangement, prompting a more uniform distribution of the cobalt phase and a reduction in pores in the sintering process, and increasing the relative density of the alloy [31,32]. Therefore, the addition of cerium facilitates the homogeneous dispersion of the cobalt phase and the densification of the alloy. The mechanism of cerium influence on the dissolution precipitation of WC during the sintering process is shown in Figure 10.

There are two methods of grain growth: one is grain aggregation growth—i.e., several grains gather together to form a large grain, but this tends to cause defects in the grain; the other is dissolution–precipitation growth—i.e., fine grain dissolution, and the dissolved W and C atoms precipitate on the surface of the coarse grain to promote grain growth. The addition of cerium elements improves the Co coating effect on the surface of the WC particles, and the contact area between the WC particles is reduced, which inhibits the growth of grains in an aggregated manner. In addition, cerium elements can promote W-dissolution, inhibit W-precipitation, and inhibit WC grains from growing in a dissolution–precipitation manner. On the other hand, the addition of cerium elements lowers the temperature of the liquid phase-formation during sintering, prolongs the liquid phase sintering time, promotes the solution precipitation process of WC, and leads to the coarsening of the WC grains. The effect of rare-earth cerium on the grain size during alloy sintering is a combination of these mechanisms [32]. At the cerium elements addition of 0~0.3 wt%, the degree of Co phase homogeneity is greatly enhanced, and the mechanism of inhibiting the grain growth is the main factor. As the cerium elements content continues to increase, the degree of Co uniformity reaches a high degree, it is difficult to continue to improve. At this time, the liquid phase sintering time is prolonged, resulting in the main factor of the mechanism of grain-coarsening. As a result, the grain size shows a trend of decreasing and then increasing with the increase in the cerium elements content. As the hardness increases as the grain size decreases, adding a certain amount of cerium can effectively increase the Rockwell hardness of cemented carbide.

Cerium oxides are present in the WC-Co phase grain boundaries and Co-bonded phase, which play a role in enriching impurity elements to purify the grain boundaries and peg them, strengthen the grain and phase boundaries [33], and thus improve the strength of the carbide. Furthermore, the addition of cerium elements inhibits the transformation of martensite, allowing more face-centered structured cobalt phases with higher plastic strain capacity to be retained in the alloy [31], which may be one of the reasons for the improved flexural strength and impact toughness.

## 5. Conclusions

This paper explores the effect of different process parameters on the chemical co-precipitation coating reaction process of WC-Co(Ce), analyzes the grain size and microstructure of WC-Co(Ce) cemented carbides with different Ce contents, and tests the mechanical properties to determine the influence law and mechanism of cerium addition on the microscopic structure and mechanical performance. The main conclusions are as follows:

(1) The optimum powder precipitation rate can be obtained when the solution temperature, precipitant addition time, and solution concentration are 50 °C, 30 min, and 0.3 mol/L, respectively. Under these process parameters, the precipitation rate of the cobalt–cerium oxalate crystal was appropriate, and the crystals of cobalt and cerium oxalate that formed were relatively small in length and particle size. The overall distribution in the solution was uniform, and the coating effect on the WC particles was good.
(2) When the cobalt content is 12%, with the addition of cerium elements, the mechanical properties of cemented carbide prepared using the chemical co-precipitation method are higher than those of cemented carbide without the addition of cerium elements. The flexural strength and impact toughness were significantly improved by adding cerium elements, which increased to 2487 MPa and 36.1 kJ/m^2^, respectively.
(3) As the concentration of CoCl_2_ in the solution increases, the accelerated co-precipitation reaction speed up the formation of cobalt–cerium oxalate nuclei, resulting in the formation of cobalt–cerium oxalate crystals without growth momentum. Then, the cobalt-cerium oxalate crystals generated by the co-precipitation reaction have a relatively small length and particle size, whose overall distribution is uniform. The addition of cerium elements is beneficial to the densification of cemented carbide and the improvement in the mechanical properties because these cerium elements can combine with impurity elements to make the cobalt phase more uniformly distributed and better encapsulated on the surface of the WC particles.
(4) The WC-Co cemented carbide prepared using the method described in this paper was optimized on the basis of the selected materials by adjusting the process parameters and cerium addition. Compared with the nanostructured cemented carbide, a small portion of the hardness and flexural strength are sacrificed to obtain higher impact toughness, while its hardness and flexural strength are stronger than that of the ultra-coarse-crystal cemented carbide, which makes it more wear-resistant and less prone to fracture while satisfying the hardness and flexural strength of the cutting and breaking of rock (such as the cutterheads of the shield tool and the pile-drilling cutterheads).

## Data Availability

Data are contained within the article.
